# Molecular Breeding for Improving Productivity of *Oryza sativa* L. cv. Pusa 44 under Reproductive Stage Drought Stress through Introgression of a Major QTL, *qDTY12.1*

**DOI:** 10.3390/genes12070967

**Published:** 2021-06-24

**Authors:** Kyaw Swar Oo, Subbaiyan Gopala Krishnan, Kunnummal Kurungara Vinod, Gaurav Dhawan, Priyanka Dwivedi, Pankaj Kumar, Prolay Kumar Bhowmick, Madan Pal, Viswanathan Chinnuswamy, Mariappan Nagarajan, Haritha Bollinedi, Ranjith Kumar Ellur, Ashok Kumar Singh

**Affiliations:** 1 Division of Genetics, ICAR-Indian Agricultural Research Institute, New Delhi 110012, India; k.swar555@gmail.com (K.S.O.); kkvinod@iari.res.in (K.K.V.); gauravbiochem2007@gmail.com (G.D.); priyankam28@gmail.com (P.D.); ky.pankaj@gmail.com (P.K.); prolay.bhowmick@icar.gov.in (P.K.B.); haritha@iari.res.in (H.B.); ranjith@iari.res.in (R.K.E.); aksingh@iari.res.in (A.K.S.); 2 Division of Plant Physiology, ICAR-Indian Agricultural Research Institute, New Delhi 110012, India; madan_physio@iari.res.in (M.P.); viswanathan@iari.res.in (V.C.); 3 Rice Breeding and Genetics Research Centre, ICAR-Indian Agricultural Research Institute, Aduthurai 612101, India; head_aduth@iari.res.in

**Keywords:** *qDTY12.1*, reproductive stage drought stress, near isogenic lines, marker assisted introgression

## Abstract

Increasing rice production is quintessential to the task of sustaining global food security, as a majority of the global population is dependent on rice as its staple dietary cereal. Among the various constraints affecting rice production, reproductive stage drought stress (RSDS) is a major challenge, due to its direct impact on grain yield. Several quantitative trait loci (QTLs) conferring RSDS tolerance have been identified in rice, and *qDTY12.1* is one of the major QTLs reported. We report the successful introgression of *qDTY12.1* into Pusa 44, a drought sensitive mega rice variety of the northwestern Indian plains. Marker-assisted backcross breeding (MABB) was adopted to transfer *qDTY12.1* into Pusa 44 in three backcrosses followed by four generations of pedigree selection, leading to development of improved near isogenic lines (NILs). Having a recurrent parent genome (RPG) recovery ranging from 94.7–98.7%, the improved NILs performed 6.5 times better than Pusa 44 under RSDS, coupled with high yield under normal irrigated conditions. The MABB program has been modified so as to defer background selection until BC_3_F_4_ to accelerate generational advancements. Deploying phenotypic selection alone in the early backcross generations could help in the successful recovery of RPG. In addition, the grain quality could be recovered in the improved NILs, leading to superior selections. Owing to their improved adaptation to drought, the release of improved NILs for regions prone to intermittent drought can help enhance rice productivity and production.

## 1. Introduction

Rice is grown in more than 100 countries across the world on a combined total of 162.06 million hectares [1]. Prominently cultivated in Asia, which accounts for about 90% of total world cultivation, rice is grown in varying ecologies ranging from upland, hill, lowland, and deep water. Grown either under irrigated or rainfed conditions, rice suffers yield loss up to 65.0% under rainfed upland and rainfed lowland ecosystems due to the intermittent occurrence seasonal drought stress. Besides, significant yield reductions occur under prolonged severe stress.

Even though rice suffers drought stress in all developmental stages, stress at the reproductive stage is particularly significant in causing yield loss. Reproductive stage drought stress (RSDS) results in shorter panicles with incomplete panicle exsertion and increased spikelet sterility due to pollen and stigma desiccation. Stress during grain filling reduces grain weight, and increases chalkiness due to poor starch granule packing. Therefore, RSDS remains a major challenge for rice production in major rice growing zones [2]. At the seedling stage, however, drought stress leads to poor seedling establishment, growth retardation, very slow growth rate, and poor tiller initiation along with reduced plant biomass development [3]. Drought stress during the vegetative growth stage, causes plant height reduction with less effective number of tillers per hill, reduced leaf area, and general stunting [4].

Rice exhibits significant heritable variation in drought response, with genotypes behaving differently in different rice ecosystems. This diversity in response is wide between the rainfed upland and irrigated lowland systems. Drought response is often related to the severity of drought, with mild stress commonly well-tolerated by most of the genotypes that are either tolerant or moderately tolerant. Severe stress although affects the yield significantly in all genotypes, drought sensitive genotypes suffers the most than the tolerant ones. Several traits have been shortlisted by the breeders as key indicators of selection for drought tolerance. Pinheiro et al. [5] identified uppermost internode elongation, flag leaf traits such as green color, carbon dioxide assimilation, chlorophyll content, and photosynthesis rate as traits significantly correlated with grain yield, panicle length, filled grain per panicle, spikelet fertility, and 1000 grain weight, making these traits indicators for selection under drought stress. Other important traits associated with drought adaptation are a deeper root system, profuse tillering, thicker wax layer, and wider leaf area. Additionally, physiological traits such as higher relative water content and high membrane stability index are used as indicators for cellular level drought tolerance [6,7].

Attempts to breed for RSDS tolerance in rice through conventional means have resulted in limited success due to poor selection efficiency for this trait, confounded by low heritability and genotype x environment interaction. However, significant progress could be achieved through molecular marker-based interventions, wherein several quantitative trait loci (QTLs) governing grain yield under drought (qDTY) have been mapped [8]. Among these, prominent QTLs such as *qDTY1.1* [9,10], *qDTY3.1* [11] and *qDTY12.1* [12] have been used for marker-assisted improvement of several elite rice cultivars that are drought sensitive. Currently, improved cultivars having qDTYs either singly or in combination have already been released in several countries [13,14].

In India, Pusa 44 is a medium- to long-duration and highly productive mega variety cultivated across the northwestern plain zone, particularly in Punjab and Haryana. Pusa 44 was developed and released by the Indian Council of Agricultural Research (ICAR)-Indian Agricultural Research Institute (IARI), New Delhi solely for the irrigated lowland ecology [15]. Catering to the livelihood of thousands of rice farmers, Pusa 44 is grown extensively in northwestern India, and is widely popular due to its high yield potential (7.0–8.0 t.ha^−1^). A non-aromatic rice variety with translucent long slender grains, Pusa 44 has semi-dwarf stature, strong culm with erect plant type, suitability to mechanical harvesting, medium duration (145–150 days), higher number of productive tillers, and superior cooking quality. However, owing to its high susceptibility to drought stress, especially for RSDS, the continuation of its cultivation has become a major topic of debate in northwestern India, where the receding groundwater level poses a serious challenge to rice production. This has led to the initiation of research to augment Pusa 44 with RSDS tolerance by targeted introgression of qDTYs, either as mono-QTL near isogenic lines (NILS) or QTL stacked NILs. There are recent reports of successful introgression of qDTYs such as *qDTY2.1* and *qDTY3.1* into Pusa 44 [15,16]. Introgressing *qDTY12.1*, a robust QTL, into Pusa 44 could also be another step toward redesigning this popular cultivar to combat RSDS. This would enable the development of improved lines with a minimum yield potential of one to two tonnes per hectare (t.ha^−1^) under severe RSDS, while ensuring the higher yield potential under irrigated lowland conditions [17]. Cultivars with potential for dual or multiple adaptation are required in the future in areas where climate is fast changing. Therefore, climate resilience will be the key attribute contributing to sustained crop production and ensuring food security under changing climate. Moreover, accelerated development of cultivars using the marker-assisted breeding strategy would be a boon over classical approaches, which normally take 10–12 years for cultivar development.

In the present study, we report the successful introgression of *qDTY12.1* into Pusa 44 through marker-assisted backcross breeding, leading to the development of improved NILs showing improved adaptation to RSDS under two contrasting environments in India.

## 2. Materials and Methods

### 2.1. Plant Materials

The popular cultivar, *Oryza sativa* L. cv. Pusa 44, which is highly sensitive to RSDS, was used as the recipient parent, the seeds of which were obtained from the Division of Genetics, ICAR-IARI, New Delhi. The donor parent for the QTL, *qDTY12.1* was IR90019-22-28-2B developed by the International Rice Research Institute from the cross, Vandana/Way Rarem [18]. All the field experiments were implemented at the research farms of ICAR-IARI at New Delhi and at the Rice Breeding and Genetics Research Centre (RBGRC), ICAR-IARI, Aduthurai, Tamil Nadu. During the development of NILs, the plants were grown with standard recommended agronomic management under irrigated ecology.

### 2.2. Markers for Selection

**Figure 1:**
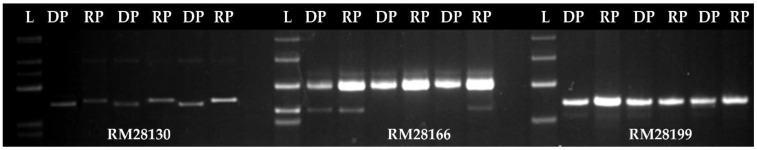
Representative gel picture showing polymorphism of foreground markers. RM28130 provided the best contrast between Pusa 44 and IR90019-22-28-2B. L: ladder; RP: recurrent parent, Pusa 44; DP: donor parent, IR90019-22-28-2B. RP and DP were replicated 3 times.

Marker-assisted selection was carried out in all generations in two steps, foreground and background. For foreground selection, initially, five peak microsatellite (SSR) markers tightly linked to *qDTY12.1* namely, RM28099, RM511, RM28130, RM28166, and RM28199 [19] were tested for polymorphism. Among these, RM28130, which provided the best contrast between parents, was further used for selection (Figure 1). Eighty-four polymorphic SSR markers identified as polymorphic from a set of 844 SSR markers across the rice genome were employed for background selection in the BC_3_F_4_ generation (Supplementary Table S1).

### 2.3. Development of Pusa 44 qDTY12.1 Near Isogenic Lines

**Figure 2:**
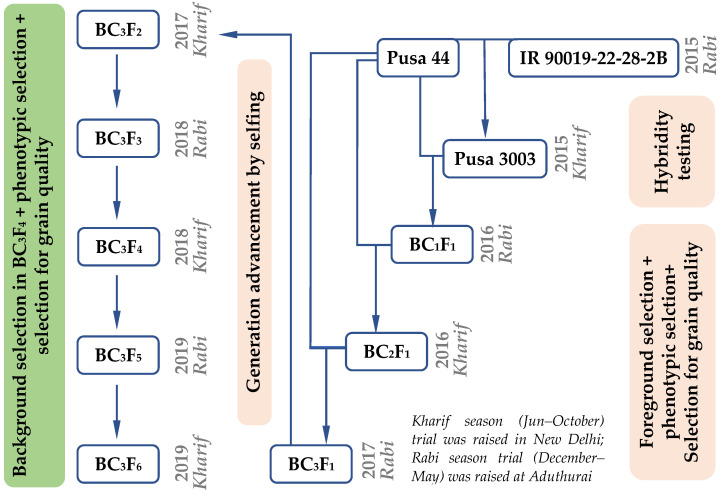
Breeding scheme adopted for the development of Pusa 44 *qDTY12.1* -NILs.

The hybridization of Pusa 44 with IR90019-22-28-2B was carried out during *Rabi* season (December–May) of 2014–2015 at Aduthurai. Pusa 44 was used as the female parent throughout the crossing program (Figure 2). The F_1_ seeds were grown in New Delhi during the next *Kharif* season (Jun–October) of 2015 and tested for hybridity using the foreground marker, RM28130. True F_1_s were backcrossed to the recipient parent, Pusa 44, to develop the BC_1_F_1_ generation. Foreground selection was carried out with BC_1_F_1_ plants and the true BC_1_F_1_s with maximum phenotypic similarity to Pusa 44 were chosen for the second round of backcrossing. The same protocol was continued until the development of BC_3_F_1_. In the BC_3_F_1_ generation, the progenies heterozygous for the target marker, RM28130 were identified and selfed to generate the BC_3_F_2_ population. Based on foreground selection in the BC_3_F_2_, plants homozygous for the donor allele were selected and subjected to phenotypic selection to identify desirable plants, which were then selfed to generate the BC_3_F_3_ generation. Further, phenotypic selection for similarity to the recurrent parent, Pusa 44 was carried out for agronomic, grain, and cooking quality traits, was carried out in all generations beginning from the BC_1_F_1_ generation. Selected BC_3_F_3_ plants were advanced to BC_3_F_4_ families by selfing. Continuing this process, improved Pusa 44 *qDTY12.1* NILs in BC_3_F_6_ with maximum of recurrent parent genome recovery (RPGR) as well as phenotypic and grain quality attributes similar to Pusa 44 were identified for further evaluation of agronomic superiority over Pusa 44 under RSDS. The RPGR was computed using the genome-wide polymorphic markers in each of the NILs as described earlier [20,21]. Additionally, recombinant selection in the carrier chromosome was also undertaken using the markers flanking *qDTY12.1* in the BC_3_F_6_ generation.

### 2.4. Field Screening under Reproductive Stage Drought Stress

Field screening of the improved NILs of Pusa 44 carrying *qDTY12.1* was carried out during two consecutive years at ICAR-IARI, New Delhi. During 2018, 7 BC_3_F_4_ lines that showed superior yield and grain quality were selected from a set of 18 lines and raised under artificially created RSDS along with the parents, Pusa 44 and IR90019-22-28-2B. The experiment was conducted in randomized complete block design with two replications under field conditions. A parallel set of the same genotypes were grown under the recommended irrigation schedule. Both treatments were raised with standard agronomic management. Both trials were grown with spacing of 20 cm × 15 cm, with each genotype occupying a 2.4 m^2^ area. In the stressed plots, drought was imposed 25 days after transplanting (50 days after sowing), and one life-saving irrigation was provided 55 days after transplanting by flood irrigation. The unstressed treatment received normal irrigation and was maintained with standing water. The moisture level in the drought stressed plots was monitored by placing tensiometers at regular intervals. At the reproductive stage, the soil moisture tension was monitored till it reached upto −70 kPa followed by lifesaving irrigation at peak stress of −70 kPa. At physiological maturity, the trials were measured for grain yield traits such as single plant yield, and plot yield as well as for agronomic traits such as plant height, number of tillers per hill, and panicle length. The remaining lines in the BC_3_F_4_ generation were grown only under irrigation. The selections from the BC_3_F_4_ lines after agronomic and grain quality evaluation were sent to RBGRC, ICAR-IARI, Aduthurai, where the lines were grown under irrigated conditions. Further, selections from this trial (Supplementary Table S1) were evaluated at ICAR-IARI, New Delhi during *Kharif* 2019, subjecting the lines under both imposed drought as well as irrigated conditions. There were 18 NILs selected under this trial, coinciding with the BC_3_F_6_ generation.

Additionally, to quantify the stress response of the NILs, stress response related indices [16,22] such as relative stress tolerance (RTOL) [23], geometric mean productivity (GMP), stress-tolerance index (STI) [24], stress susceptibility index (SSI) [25], harmonic mean [26], yield index (YI) [27], and yield stability index [28], were computed for comparative evaluation.

### 2.5. Grain Quality Analysis

The grain quality of the Pusa 44 *qDTY12.1* -NILs was tested under both stressed and unstressed conditions to check if the drought has any influence on the quality parameters. For this, grains were tested for hulling and milling recovery and cooking parameters such as grain dimensions (length and width) before and after cooking, length to width ratio, as well as kernel elongation ratio on cooking.

### 2.6. Statistical Analyses

All the data recorded from the replicated trials were subjected to analysis of variance (ANOVA) for all the agronomic and grain quality traits. Mean comparison was carried out between the irrigated control and drought stress screening plots.

## 3. Results

### 3.1. Marker Polymorphism between the Parents

To identify markers for foreground selection, five SSR markers linked to the QTL *qDTY12.1*, namely, RM28099, RM511, RM28130, RM28166, and RM28199, were screened for parental polymorphism; of which, RM28130 showed better resolution between parents, making it the ideal choice for further foreground selections. All other markers were found to be monomorphic between parents. RM28130 produced an amplicon size of 176 bp in the donor parent, while the amplicon size in the recurrent parent, Pusa 44, was 180 bp. For background selection, parental polymorphism was carried out using 844 genome-wide SSR markers. The background genomes of the parents were found to be diverse for 84 markers, indicating a diversity of 10.0% between them (Table 1). Chromosome-wise genome-wide polymorphism indicated that chromosome 3 showed the maximum diversity of 19.4%, and chromosome 1 was the least polymorphic (5.4%). Chromosome 12, the carrier chromosome of *qDTY12.1*, had a polymorphism of 10.8%. Among the 12 chromosomes, as many as six showed a diversity above 10%.

### 3.2. Development of NILs through Marker Assisted Breeding

**Figure 3:**
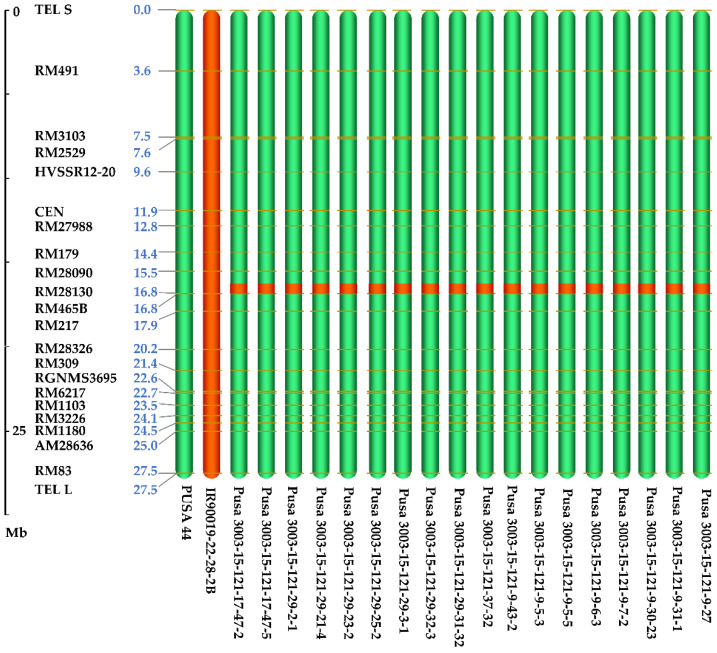
Graphical genotype of Chromosome 12, the carrier chromosome of the QTL, *qDTY12.1* among the Pusa44 *qDTY12.1*-NILs. 29 NILs showed complete recovery of Chromosome 12, based on the polymorphic flanking markers of the QTL.

The hybridization of parents Pusa 44 and IR90019-22-28-2B was attempted during *Rabi* 2014–2015 season at the offseason nursery at RBGRC, ICAR-IARI, Aduthurai to generate 200 F_1_ seeds. The cross was named Pusa 3003. A total of 106 F_1_ plants were raised at ICAR-IARI, New Delhi during *Kharif* 2015 and were subjected to hybridity testing using the foreground marker, RM 28130, at the active tillering stage (Table 2). Thirty-six true F_1_ plants that showed clear heterozygous banding for RM28130 were tagged for backcrossing with the recurrent parent, Pusa 44. The BC_1_F_1_ seeds generated from one of the F_1_ plants were raised in *Rabi* 2015–2016 at Aduthurai. Foreground selection was carried out among 18 BC_1_F_1′_s to identify five plants heterozygous for the *qDTY12.1* linked marker RM28130, which were subjected to phenotypic selection. The plants with maximum phenotypic similarity to Pusa 44 were used for generating the BC_2_F_1_ seeds. Of the 72 BC_2_F_1_ plants raised during the *Kharif* 2016 at New Delhi, 31 were selected based on the foreground and phenotypic selection for further backcrossing with Pusa 44. Thirty BC_3_F_1_s were raised during *Rabi* 2016–2017 at Aduthurai. Foreground selection among the BC_3_F_1_ plants could identify seven plants heterozygous for the QTL linked SSR marker RM28130, and higher phenotypic resemblance with Pusa 44, after rigorous phenotypic selection. The selection parameters included visual assessment of plant statures, such as plant height, tillering habit, grain morphology, panicle architecture, and grain yield. All the selected plants were selfed during the flowering stage and harvested individually. In *Kharif* 2017, six BC_3_F_2_ populations derived from the best BC_3_F_1_ plants were raised at New Delhi. Among these, 67 plants were selected from across the six families by foreground selection, wherein only plants with homozygous donor (IR90019-22-28-2B) alleles (176 bp) were selected for generation advancement. The 67 BC_3_F_3_ families were grown at Aduthurai during the *Rabi* 2017–2018, from which progenies similar to Pusa 44 were shortlisted for further evaluation of agronomic performance, resulting in the selection of 18 plants. All 18 selected lines carried the donor allele for *qDTY12.1* in the homozygous state. Subsequently, the selected lines were raised during *Kharif* 2018 at New Delhi and subjected to phenotypic and background selections. All 84 polymorphic markers were used for background selection in 18 NILs, which originated from five BC_3_F_3_ families. During *Rabi* 2018–2019, the BC_3_F_5_ generation was raised at Aduthurai, where further phenotypic selection for agro-morphological as well as grain and cooking quality was carried out to identify six potential NILs for testing. All the six NILs were subsequently evaluated during *Kharif* 2019 in New Delhi. The RPGR of the NILs ranged between 94.7 and 98.7%. By the BC_3_F_6_ generation, the recovery on chromosome 12 was 100% in as many as 29 NILs out of 41 tested, as determined by the flanking markers (Figure 3).

### 3.3. Agronomic Evaluation for Differential Response under Stressed and Unstressed Treatments

Evaluation of the NILs for agronomic traits under irrigated and imposed drought conditions for two consecutive years revealed significant variation in components such as genotype and genotype x treatment for most of the traits (Table 3), except for days to 50% flowering and effective tiller number. During 2018, the genotypic variation for grain yield was not apparent, but between treatments the variation was remarkable. During 2019, however, all three components showed clear variation for grain yield.

### 3.4. Drought Response of Promising Pusa 44 qDTY12.1-NILs in BC_3_F_4_

Compared to the unstressed control, the recurrent parent, Pusa 44, showed significant delay in flowering, with reduced plant height, tiller number, and grain yield under drought stress (Table 4). The donor parent, IR90019-22-28-2-B, expressed a striking improvement in grain yield compared to other traits such as plant height, panicle length, and tiller number. The yield reduction in Pusa 44 was 9.5 times lower than when irrigated, whereas in IR90019-22-28-2-B it was only about 1.5 times lower than when irrigated. On the other hand, NILs showed a yield reduction level almost similar to that of the donor parent under drought stress conditions, while yielding similarly to Pusa 44 under irrigated conditions, except that five NILs had significantly lower yields under irrigated control. The ratio between unstressed yield to stressed yield among the NILs ranged from 1.9 (Pusa 3003-15-121-9-4) to 3.6 (Pusa 3003-15-121-29-23).

The highest grain yield under drought among the NILs was recorded in Pusa 3003-15-121-30-23 (327.39 g.sqm^−1^), and all the other NILs were at par with the highest yielding NIL under drought stress. Under irrigated condition, all the NILs, except for Pusa 3003-15-121-9-4, and Pusa 3003-15-121-9-27 were at par with Pusa 44 for grain yield. For other traits, there was no significant variation among the NILs both under stressed and unstressed conditions, except for reduced plant height in Pusa 3003-15-121-31-1 in the unstressed plots.

### 3.5. Agronomic Performance of BC_3_F_5_ Generation NILs under Irrigation

The NILs selected in BC_3_F_4_ (including those tested under drought as well as those tested only under irrigated conditions in New Delhi) were evaluated at RBGRC, Aduthurai during *Rabi* 2018–2019. The data on agronomic performance (Supplementary Table S1) indicated that all NILs had statistically similar performance for agronomic traits such as days to 50% flowering, plant height, effective tillers number per hill, filled grains per panicle, total number and fertility of spikelets, and grain yield. However, in some of the NILs, the 1000 grain weight was higher than that of Pusa 44. Average grain yield of Pusa 44 was 402.7 g.sqm^−1^, while NILs on average produced a grain yield of 384.0 g.sqm^−1^.

### 3.6. Response of BC_3_F_6_ Selections under Stressed and Unstressed Conditions

The agronomic performance of the Pusa 44 NILs in the BC_3_F_6_ generation, both under stressed and unstressed conditions, is presented in Table 5.

In general, under unstressed treatment, the NILs demonstrated performance at par with Pusa 44, by showing statistically indifferent measurements for most of the agronomic traits. However, agronomic performance of the NILs significantly deviated from that of Pusa 44, particularly for the grain yield. Nevertheless, within each treatment, traits such as days to 50% flowering, plant height, tiller number, panicle length, etc. did not show much variation. Under drought, the days to 50% flowering, however, showed a mixed pattern as one of the parents (Pusa 44) had a delay in flowering under stress, while the other parent had a tendency to flower early under stress. There were nine NILs that showed earliness in flowering under drought, while another nine showed delayed flowering behavior. Among the NILs that showed earliness in flowering, Pusa 3003-15-121-17-47-2 flowered 9.5 days earlier than its irrigated system behavior. This was followed by the NIL, Pusa 3003-15-121-17-47-5, which exhibited an earliness of 4.5 days compared to that under unstressed control. However, in the recurrent parent, the delay in flowering under stress was 11.5 days, whereas the donor parent had closely similar flowering times under both conditions, with a subtle tendency to flower early with a 1.5 days advantage. Grain yields under drought among the NILs varied between 95.0 g.sqm^−1^ and 295.1 g.sqm^−1^, but under unstressed conditions, ranged from 589.5 g.sqm^−1^ to 910.3 g.sqm^−1^. However, the NILs that produced higher grain yields under irrigation as well as drought were Pusa 3003-15-121-9-6-3, Pusa 3003-15-121-30-23, Pusa 3003-15-121-17-47-2, and Pusa 3003-15-121-9-27 followed by Pusa 3003-15-121-31-1. Among the genotypes tested, the recurrent parent, Pusa 44, was the one that was most affected by drought, whereas the donor parent, IR90019-22-28-2-B, was the least affected. Comparing the ratio between grain yields under unstressed and stressed plots, the largest ratio was shown by the NIL, Pusa 3003-15-121-29-3-1 (8.1), followed by Pusa 44, which had a reduced yield under stress that was eight times lower than its irrigated system yield.

### 3.7. Indicators of Drought Tolerance among the Pusa 44 qDTY12.1 -NILs

A set of drought response indicators were calculated for assessing the drought tolerance of the NILs in relation to their parents (Table 6). The cumulative tolerance of each NIL was computed using the average ranks of all the indices. The highest average of 19.1 was recorded for the recurrent parent, Pusa 44 while the donor, IR90019-22-28-2-B recorded a rank average of 4.0. Among the NILs, there were three, Pusa 3003-15-121-17-47-2, Pusa 3003-15-121-9-27, and Pusa 3003-15-121-31-1, that had better average ranks than the donor, while an additional six NILs had average ranks lower than 10.5, the median average rank. Comparing the absolute values for grain yield and average rank, it was found that the yield under stress conditions was highly correlated with a coefficient of −0.958, whereas the yield under irrigated conditions correlated with a coefficient of 0.262. Furthermore, the grain yield of the better ranking lines under drought conditions was found to range between 158.9 and 295.1 g.sqm^−1^, whereas their unstressed yield ranged from 592.45 to 847.6 g.sqm^−1^.

### 3.8. Grain Quality of Pusa 44 qDTY12.1 Near Isogenic Lines

The grain quality of the Pusa 44 *qDTY12.1* -NILs developed in this study, in BC_3_F_6_ generation is presented in Table 7. The parents, Pusa 44 and IR90019-22-28-2B, had uncooked grain lengths of 6.83 and 6.53 mm, respectively, which were statistically at par. Similarly, all the NILs possesses uncooked grain lengths statistically similar with the values for both parents. Additionally, their grain width and the length-width ratios were also found to be non-significantly different from one other. A parallel pattern was observed for grain traits such as length and width when cooked. Nevertheless, only marginal variations between the parents and NILs were observed for grain processing properties such as hulling percentage and milling percentage.

## 4. Discussion

As hypothesized in this study, future rice cultivars should possess multiple stress tolerance combined with higher yield and desirable grain quality. Such resilient varieties will gain popularity among farmers, as they will contribute to better harvest yields in the event of a climatic anomaly. One of the foremost targets for abiotic tolerance is endurance under RSDS. There are several yield contributing QTLs identified for RSDS tolerance in rice, such as *qDTY12.1*, which was established to work under varying genetic backgrounds [29]. Selection of a consistent QTL is very important to the ultimate success of the backcross breeding program, because introgression of unreliable QTLs/genes may result in considerable losses in time and money. Equally important is the selection of parental lines, because the improved lines are to be used for quick replacement of existing sensitive cultivars, and should enable ready adoption by farmers. In addition, care should be exercised in the choice of the donor, because the ultimately improved line should not carry any undesirable trait from the donor, only the trait desired through introgression. Among undesirable traits, grain quality is a complex of multiple factors that can be affected when the donor is inferior in grain quality. In practical molecular breeding, stringent phenotypic selection has been recognized as a key criterion for imparting all the desirable qualities such as grain quality [21,30].

Pusa 44 was selected as the recurrent parent because it has several desirable features such as amenability to mechanical harvest, easily processable grains, and excellent cooking characteristics for consumption. The donor, IR90019-22-28-2-B, which carries the *qDTY12.1* allele, was a derivative of Vandana, an early duration cultivar popular in eastern India. The donor line carried *qDTY12.1* from Way Rarem, an Indonesian cultivar known for its susceptibility to drought [29,31]. Mapped earlier in an F_3_ population of Vandana/Way Rarem [12], *qDTY12.1* has been identified as a major QTL contributing as much as 51% of the phenotypic variation for yield under RSDS. This QTL could improve the drought tolerance of an already tolerant cultivar such as Vandana, and is effective under varying genetic backgrounds adapted to different ecosystems such as upland and lowland [31]. When present, *qDTY12.1* is known to increase tillering, increase in biomass, reduce crop duration, and improve the harvest index [32]. Furthermore, its prevalence among 85% of the random drought cultivars is remarkable, and evidence indicates that other cereal genomes carry homologous sequences to this QTL [33]. Due to its versatility, *qDTY12.1* has been a choice QTL for introgression into multiple backgrounds across the globe for improved RSDS tolerance [14] and is known to integrate high grain yields under mild to severe stress conditions.

We employed MABB for introgression of *qDTY12.1* into Pusa 44. MABB has been established as a successful approach to the introgression of target genes/QTLs, particularly in rice, with high precision, very good success rates, and a short developmental period [34]. This can be construed from the fact that, within the decade from 2008 to 2018, eight varieties were developed and released by ICAR-IARI using the MABB approach [30]. Additionally, several other improved lines targeting multiple features are also under development. Among these, Pusa 44 has been a prominent choice among the non-Basmati rice varieties for improvement due to the advantages described earlier. Recent developments in the improvement of Pusa 44 by the introgression of QTLs imparting RSDS tolerance such as *qDTY2.1* and *qDTY3.1* were reported by Dwivedi et al. [15] and Oo et al. [16].

Throughout the developmental process for Pusa 44 *qDTY12.1* -NILs, we performed foreground selection in all generations but resorted to phenotype selection up until the BC_3_F_4_ generation. This has been a conspicuous deviation from our earlier approaches wherein background selection was augmented with phenotypic selection to ensure better RPG as well as recurrent parent phenome (RPP) recovery [34,35,36]. Ultimately, deferred background selection provided a recovery of 94.7–98.7%, closely similar to the level of recovery that was previously reported [37,38]. This provided us with two advantages: (i) considerable time could be saved on screening the background markers, which helped us to shuttle the breeding materials between New Delhi and Aduthurai in time, and (ii) considerable resources could be saved as the background recovery was tested on a small panel of selected lines at BC_3_F_4_. Although the approach resulted in gaining the desired RPGR, the uncertainty of gain in RPG remained until the final background selection. This is feasible only when the breeder has excellent expertise in phenotypic selection. Another noticeable feature has been the complete recovery of the target chromosome 12, among several of the NILs.

The NILs generated in this study performed well agronomically under imposed drought stress as well as unstressed conditions. Evaluated for two years during the *Kharif* seasons of 2018 and 2019, the NILs showed significant yield reduction under drought stress when compared to yields under unstressed, irrigated conditions. A similar reduction in yield under imposed drought conditions was reported among the qDTY introgressed NILs of mega cultivars such as Pusa Basmati 1 [39] and Pusa 44 [15,16]. However, the level of yield reduction was significantly lower among the NILs, with 6.5 times higher yields than those with the recurrent parent, Pusa 44. On average, the yield under drought conditions among the NILs was 2.3 times lower than that under unstressed conditions. For comparison, in Pusa 44 the yield due to RSDS was 9.7 times lower than the irrigated yield. This indicated that the *qDTY12.1* introgression could manifest consistent and discernible drought tolerance among the improved NILs. Considering the intensity of drought imposed, up to a significant soil moisture tension of −70 kPa, we concluded that the level of tolerance achieved was significant. Ghosh and Singh [40] reported that in aerobic rice, soil moisture tension of −60 kPa could result in a yield loss of 42.8% and concluded that −40 kPa could be used as the threshold for scheduling irrigation.

**Figure 4:**
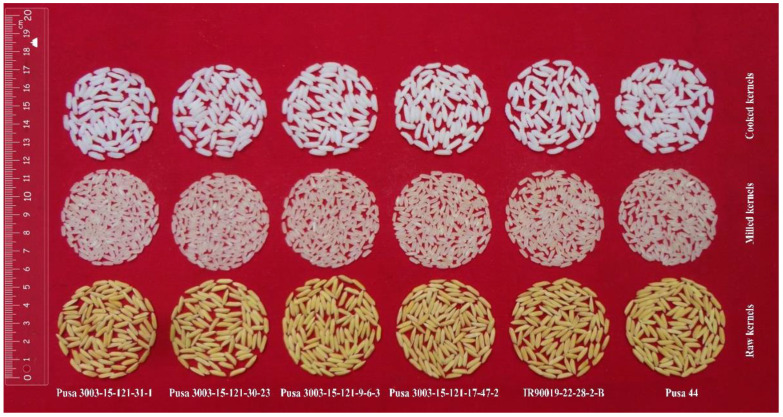
Grain morphology of the selected Pusa 44 *qDTY12.1* -NILs, showing remarkable recovery of grain and cooking quality.

The remarkable gain in yield response in the Pusa 44 derived *qDTY12.1* -NILs could not be attributed to component traits. Comparing agronomic performance across years and between stressed and unstressed treatments, the only trait that showed striking variation was plant height. Plant height was, however, found to decrease under drought in all the genotypes, including the recurrent as well as donor parents. However, a distinguishable pattern in the reduction in height could not be drawn between the sensitive parent, Pusa 44, and the NILs. This could imply that the effect of *qDTY12.1* in imparting drought endurance could vary between the genetic backgrounds. Similar differences in the agronomic advantage of *qDTY12.1* have already been reported [41]. When compared to agronomic changes that are conditioned by different qDTY QTLs in rice, we could see that plant height reduction was a common response under drought conditions, as reported in previous works, wherein *qDTY2.1* and *qDTY3.1* were introgressed into Pusa 44 [15,16] and Pusa Basmati 1 [39]. In addition, a delay in flowering and a reduction in the number of tillers were also reported in the improved NILs. Interestingly, in the present case, the Pusa 44 *qDTY12.1* NILs did not show any significant delay in flowering or reduction in tiller numbers. These observations provide possible support to the earlier reports that *qDTY12.1* improved tillering and flowering response in rice [29]. Furthermore, a remarkable recovery of grain quality in terms of cooking properties, grain dimensions, and processing properties has been realized among the Pusa 44 *qDTY12.1* lines (Figure 4).

The present investigation identified some of the outstanding NILs, with potential for improved yields under both drought and irrigated environments. NILs such as Pusa 3003-15-121-17-47-2, Pusa 3003-15-121-9-27, Pusa 3003-15-121-31-1, Pusa 3003-15-121-30-23, and Pusa 3003-15-121-9-6-3 have better drought response indices when compared to the recurrent parent, Pusa 44. Drought response indices used in this study placed significantly more weight on drought response than yield under normal management. This was evident from the strong association between the average rank and yield under drought conditions (−0.958). The correlation to the yield under irrigated conditions was 0.262. This provided us with the opportunity to select the best drought-tolerant NIL based on the lowest average rank. In breeding for drought tolerance, selection of the line with the best drought tolerance should be given preference over yield potential in irrigated environments to ensure reasonable grain yield in the event of a severe RSDS episode.

Grain quality remains an integral goal of crop improvement, especially for a crop such as rice, irrespective of the target trait of introgression as rice is consumed with minimal processing. Desirable grain quality in the improved NILs was accomplished through rigorous selection for quality components during the breeding process. It is noteworthy that the processing properties of the improved lines did not deteriorate under stressed treatment. However, a slight reduction in grain dimensions was noticed among the NILs, similarly to the reduction noticed in Pusa 44. A similar reduction in grain dimensions was noticed by Dhawan et al. [39] among the Pusa Basmati 1 *qDTY1.1* NILs. Desired grain quality in the selected NILs would facilitate their ready adoption by farmers who are already familiar with the grain quality of Pusa 44, augmented by the potential of yielding well under RSDS.

## 5. Conclusions

Through this study, we were able to establish a modified MABB approach with background selection deferred, allowing successful introgression of *qDTY12.1* without any undesired effects. The improved grain yield under drought conditions in the NILs corroborated the findings of earlier reports that emphasized compatibility with wider genetic backgrounds belonging to either lowland or upland ecosystems [32]. We presume that the addition of this QTL could leverage the adaptation of Pusa 44 to the changing dynamics of water availability for rice production in northwestern India. That assessment notwithstanding, the consistent yield performance of the improved NILs qualifies them as candidates for pan-India multi-location testing under the all-India coordinated rice improvement program. As the improved Pusa 44 *qDTY12.1* -NILs is in an elite background, they can be used also as donors for future RSDS improvements using diverse cultivar backgrounds. The enhanced tolerance of the improved NILs to RSDS may facilitate their adoption elsewhere in the country beyond current niche districts: for example, in eastern India, where intermittent episodes of RSDS are a regular feature. We believe that the development of these NILs could be a step forward in achieving climate resilience in future rice cultivars.

## Figures and Tables

**Table 1:** Chromosome wise polymorphism between the parents, Pusa 44 and IR90019-22-28-2B.

Chromosome	Markers Used			Diversity (%)
	Polymorphic	Monomorphic	Total	
1	7	122	129	5.43
2	11	56	67	16.42
3	7	29	36	19.44
4	5	76	81	6.17
5	6	84	90	6.67
6	9	62	71	12.68
7	7	34	41	17.07
8	7	94	101	6.93
9	5	31	36	13.89
10	4	67	71	5.63
11	9	47	56	16.07
12	7 *	58	65	10.77
Total	84	760	844	9.95

* The foreground marker, RM28130, is not included in the list of markers used for background selection across chromosome 12.

**Table 2:** Progressive selection statistics for Pusa 44 *qDTY12.1* near isogenic lines.

Generation	No. of Plants/Lines		Selection Method	RPGR %
	Raised	Selected		
F_1_	106	36	Hybridity testing	50%
BC_1_F_1_	18	5	FS + PS	-
BC_2_F_1_	72	31	FS + PS	-
BC_3_F_1_	30	7	FS + PS	-
BC_3_F_2_	6 (Families)	67	FS + PS	-
BC_3_F_3_	67 (Lines)	18 *	FS + PS	-
BC_3_F_4_	18 (Lines)	18 NILs	PS + BS + GQ	94.7–98.7
BC_3_F_5_	18 NILs	6 NILs **	PS + BS + GQ	94.7–98.7

FS, foreground selection; BS, background selection; PS, phenotypic selection; GQ, selection for grain and cooking qualities; NIL, near isogenic line; * 18 plants belonged to 5 families and 15 lines; ** 6 NILs belonged to 4 families and 6 lines.

**Table 3:** Combined analysis of variance among the Pusa 44 NILs carrying *qDTY12.1* under irrigated (US) and drought stress (S) during *Kharif* 2018 and *Kharif* 2019.

Traits	Treatment	Genotype	Genotype x Treatment	CV%	CD (5%)
Days to 50% flowering	10.51 ^ns^	42.92 **	15.48 **	2.82	10.03
Plant height	6656.87 **	89.60 **	29.95 **	2.94	8.49
Effective tillers per hill	0.007 ^ns^	6.37 *	6.94 **	12.46	6.58
Panicle length	50.99 **	3.91 **	1.98 **	3.48	2.93
Plot yield *Kharif* 2018	1341072.08 **	10927.24 ^ns^	21542.61 **	4.33	44.22
Plot yield *Kharif* 2019	6174952.63 **	10872.42 **	23166.12 **	11.42	110.02

*, ** Significant at 0.05 and 0.01 probability level, respectively; ns, non-significant; CV, coefficient of variation; CD, the critical difference at 5% level probability, by Tukey’s honestly significant test.

**Table 4:** Agronomic performance of Pusa 44 *qDTY12.1*-NILs under stressed and unstressed conditions during *Kharif* 2018 in New Delhi.

Line	DFF (Days)		Ht (cm)		CmN		PnL (cm)		Yld (g.sqm^−1^)		
	S	US	S	US	S	US	S	US	S	US	rPLY
Pusa 3003-15-121-30-14	91.5 ^ab^	91.5 ^b^	69.1	77.6 ^ab^	8.9 ^b^	9.6 ^b^	22.3	21.9 ^ab^	295.2 ^a^	716.9 ^ab^	2.4
Pusa 3003-15-121-30-23	91.0 ^ab^	91.5 ^b^	69.5	74.0 ^ab^	7.9 ^b^	8.1 ^b^	22.0	21.3 ^b^	327.4 ^a^	707.5 ^abc^	2.2
Pusa 3003-15-121-31-1	95.0 ^ab^	95.5 ^ab^	66.8	62.3 ^c^	9.0 ^b^	9.3 ^b^	21.4	20.4 ^b^	288.9 ^a^	730.1 ^a^	2.5
Pusa 3003-15-121-29-23	83.0 ^b^	86.5 ^b^	77.3	78.1 ^a^	7.9 ^b^	8.8 ^b^	20.3	22.4 ^ab^	198.7 ^ab^	708.8 ^abc^	3.6
Pusa 3003-15-121-9-1	92.0 ^ab^	93.0 ^ab^	67.5	73.4 ^abc^	7.4 ^b^	7.5 ^b^	22.8	20.1 ^b^	297.7 ^a^	601.9 ^abc^	2.0
Pusa 3003-15-121-9-4	91.5 ^ab^	95.5 ^ab^	65.4	71.0 ^abc^	6.4 ^b^	8.1 ^b^	20.6	20.4 ^b^	301.7 ^a^	576.0 ^c^	1.9
Pusa 3003-15-121-9-27	92.0 ^ab^	92.0 ^ab^	72.3	76.4 ^ab^	8.8 ^b^	9.4 ^b^	22.1	22.0 ^ab^	249.8 ^a^	587.7 ^bc^	2.4
Pusa 44	96.0 ^ab^	106.0 ^a^	73.0	75.9 ^ab^	7.3 ^b^	7.9 ^b^	22.1	20.9 ^b^	75.2 ^b^	732.9 ^a^	9.8
IR90019-22-28-2-B	102.0 ^a^	103.5 ^a^	65.1	67.6 ^bc^	16.7 ^a^	17.0 ^a^	22.5	27.4 ^a^	290.6 ^a^	437.2 ^d^	1.5
CD (5%)	12.8	12.0	ns	11.7	6.7	6.9	ns	5.7	148.3	136.4	
CV%	3.4	3.1	5.7	5.1	18.5	17.7	4.5	6.4	14.1	5.2	

DFF, days to 50% flowering; Ht, plant height; CmN, tiller (culm) number; PnL, panicle length; Yld, plot yield; rPLY, the ratio between unstressed and stressed yield (reciprocal of yield stability index); ns, non-significant; CV%, coefficient of variation; CD, critical difference; S, stressed; US, unstressed. Means followed by similar letters are not statistically significant at 5% level, based on Tukey’s honestly significant test.

**Table 5:** Agronomic characteristics of Pusa 44 *qDTY12.1*-NILs under stressed and unstressed conditions during *Kharif* 2019 in New Delhi.

Pedigree	DFF		PHT		TLN		PNL		PLY			RPGR (%)
	S	US	S	US	S	US	S	US	S	US	rPLY	
Pusa 3003-15-121-17-47-2	82.0 ^d^	92.5 ^b^	82.7 ^a^	84.4 ^b–e^	18.7 ^a^	10.8	21.6 ^ab^	20.8 ^d^	295.1 ^a^	799.1 ^ab^	2.7	97.4
Pusa 3003-15-121-17-47-5	89.5 ^bcd^	93.0 ^b^	73.4 ^abc^	88.4 ^a–d^	13.8 ^ab^	13.6	21.2 ^ab^	21.8 ^bcd^	176.7 ^de^	847.6 ^ab^	4.8	97.4
Pusa 3003-15-121-29-2-1	93.0 ^bcd^	93.0 ^b^	61.0 ^de^	80.7 ^def^	14.2 ^ab^	14.3	21.2 ^ab^	23.6 ^bc^	141.8 ^efg^	589.5 ^bc^	4.2	98.7
Pusa 3003-15-121-29-21-4	95.0 ^bc^	96.5 ^ab^	61.5 ^de^	80.9 ^def^	14.6 ^ab^	13.1	22.3 ^ab^	23.7 ^b^	121.7 ^fgh^	743.2 ^ab^	6.1	98.7
Pusa 3003-15-121-29-23-2	91.5 ^bcd^	94.0 ^b^	63.9 ^cd^	83.2 ^cde^	13.6 ^ab^	15.3	21.4 ^ab^	22.0 ^bcd^	158.9 ^ef^	707.6 ^abc^	4.5	98.7
Pusa 3003-15-121-29-25-2	89.0 ^cd^	93.5 ^b^	72.2 ^bc^	88.2 ^a–d^	12.6 ^ab^	11.6	21.6 ^ab^	22.2 ^bcd^	178.2 ^de^	774.0 ^ab^	4.3	98.7
Pusa 3003-15-121-29-3-1	88.5 ^cd^	93.0 ^b^	64.2 ^cd^	87.2 ^b–e^	13.0 ^ab^	11.9	21.6 ^ab^	24.1 ^b^	106.3 ^gh^	862.2 ^ab^	8.1	98.7
Pusa 3003-15-121-29-32-3	92.0 ^bcd^	91.5 ^b^	67.8 ^bcd^	96.1 ^a^	10.2 ^b^	15.3	19.8 ^ab^	23.5 ^bc^	107.5 ^gh^	752.7 ^ab^	7.0	97.4
Pusa 3003-15-121-31-32	90.5 ^bcd^	91.0 ^b^	65.4 ^bcd^	84.8 ^b–e^	12.6 ^ab^	13.6	19.6 ^b^	22.9 ^bcd^	124.5 ^fgh^	910.3 ^a^	7.3	98.7
Pusa 3003-15-121-9-37-2	95.0 ^bc^	93.5 ^b^	68.6 ^bcd^	87.2 ^b–e^	11.9 ^b^	15.2	20.9 ^ab^	21.9 ^bcd^	140.8 ^efg^	824.5 ^ab^	5.9	97.4
Pusa 3003-15-121-9-43-2	93.0 ^bcd^	92.5 ^b^	65.4 ^bcd^	84.9 ^b–e^	12.8 ^ab^	12.0	20.8 ^ab^	22.2 ^bcd^	114.0 ^fgh^	776.5 ^ab^	6.8	94.7
Pusa 3003-15-121-9-5-3	91.5 ^bcd^	92.0 ^b^	65.5 ^bcd^	84.0 ^b–e^	13.5 ^ab^	14.7	21.1 ^ab^	23.3 ^bcd^	95.0 ^h^	734.7 ^abc^	7.7	97.4
Pusa 3003-15-121-9-5-5	98.0 ^bc^	93.0 ^b^	67.1 ^bcd^	86.4 ^b–e^	13.8 ^ab^	14.4	22.3 ^ab^	21.9 ^bcd^	105.7 ^gh^	694.6 ^abc^	6.6	97.4
Pusa 3003-15-121-9-6-3	91.5 ^bcd^	92.5 ^b^	74.5 ^ab^	92.2 ^ab^	13.6 ^ab^	12.0	22.0 ^ab^	24.2 ^b^	207.7 ^cd^	843.7 ^ab^	4.1	97.4
Pusa 3003-15-121-9-7-2	95.0 ^bc^	93.0 ^b^	64.9 ^bcd^	85.8 ^b–e^	13.3 ^ab^	12.9	21.5 ^ab^	23.9 ^b^	178.2 ^de^	775.2 ^ab^	4.4	94.8
Pusa 3003-15-121-30-23	97.5 ^bc^	92.5 ^b^	67.4 ^bcd^	86.6 ^b–e^	14.7 ^ab^	11.9	21.4 ^ab^	22.9 ^bcd^	235.7 ^bc^	771.5 ^ab^	3.3	98.7
Pusa 3003-15-121-31-1	100.5 ^bc^	94.0 ^b^	53.7 ^e^	72.9 ^f^	14.8 ^ab^	16.6	19.6 ^b^	21.1 ^cd^	283.6 ^a^	592.5 ^bc^	2.1	97.4
Pusa 3003-15-121-9-27	98.0 ^b^	97.5 ^ab^	66.5 ^bcd^	80.4 ^def^	14.6 ^ab^	17.3	21.6 ^ab^	22.7 ^bcd^	267.3 ^ab^	714.2 ^abc^	2.7	98.7
Pusa 44	116.5 ^a^	105.0 ^ab^	69.3 ^bcd^	79.7 ^ef^	14.8 ^ab^	14.9	23.4 ^a^	23.2 ^bcd^	93.3 ^h^	743.1 ^ab^	8.0	-
IR90019-22-28-2-B	102.0 ^b^	103.5 ^a^	65.1 ^bcd^	90.8 ^abc^	16.7 ^ab^	17.0	22.5 ^ab^	27.4 ^a^	315.7 ^a^	473.5 ^c^	1.5	-
CV%	3.4	2.1	3.6	2.4	11.7	13.2	4.2	2.7	6.5	10.0		
CD (5%)	12.9	8.0	9.8	8.4	6.6	ns	3.6	2.6	45.1	301.8		

DFF, days to 50% flowering; PHT, plant height; TLN, tillers number hill; PNL, panicle length; PLY, plot yield; rPLY, ratio between unstressed and stressed yield (reciprocal of yield stability index); ns, non-significant; CV%, coefficient of variation, CD, critical difference; S, stressed; US, unstressed. Means followed by similar letters are not statistically significant at 5% level, based on Tukey’s honestly significant test.

**Table 6:** Drought tolerance indices for Pusa 44 *qDTY12.1* near isogenic lines based on the performance in *Kharif* 2019.

Pedigree	Drought Response Indices							Average Rank
	RTOL	GMP	SSI	STI	HAM	YI	YSI	
Pusa 3003-15-121-17-47-2	62.8 ^d^	485.3 ^a^	0.81 ^cd^	0.43 ^a^	430.6 ^a^	1.73 ^a^	0.37 ^bc^	2.0
Pusa 3003-15-121-17-47-5	79.0 ^ab^	386.3 ^abc^	1.02 ^ab^	0.27 ^b–e^	291.9 ^cd^	1.03 ^de^	0.21 ^cde^	8.7
Pusa 3003-15-121-29-2-1	75.9 ^abc^	289.0 ^cde^	0.98 ^abc^	0.15 ^de^	228.5 ^d–g^	0.83 ^efg^	0.24 ^cde^	10.9
Pusa 3003-15-121-29-21-4	83.6 ^ab^	300.7 ^cde^	1.08 ^ab^	0.16 ^de^	209.1 ^efg^	0.71 ^fgh^	0.16 ^de^	13.3
Pusa 3003-15-121-29-23-2	77.6 ^abc^	335.3 ^b–e^	1.00 ^abc^	0.20 ^b–e^	259.5 ^de^	0.92 ^ef^	0.22 ^cde^	10.3
Pusa 3003-15-121-29-25-2	76.9 ^abc^	371.3 ^bcd^	1.00 ^abc^	0.25 ^b–e^	289.6 ^cd^	1.04 ^de^	0.23 ^cde^	8.0
Pusa 3003-15-121-29-3-1	87.7 ^a^	302.6 ^cde^	1.14 ^a^	0.16 ^cde^	189.2 ^efg^	0.62 ^gh^	0.12 ^e^	16.7
Pusa 3003-15-121-29-32-3	85.8 ^a^	284.3 ^de^	1.12 ^a^	0.15 ^de^	188.0 ^efg^	0.63 ^gh^	0.14 ^e^	16.1
Pusa 3003-15-121-31-32	86.2 ^a^	336.3 ^b–e^	1.12 ^a^	0.20 ^b–e^	218.8 ^d–g^	0.73 ^fgh^	0.13 ^e^	13.9
Pusa 3003-15-121-9-37-2	82.9 ^ab^	340.6 ^b–e^	1.08 ^ab^	0.21 ^b–e^	240.4 ^def^	0.82 ^efg^	0.17 ^de^	11.3
Pusa 3003-15-121-9-43-2	85.2 ^a^	297.0 ^cde^	1.10 ^ab^	0.16 ^de^	198.5 ^efg^	0.67 ^fgh^	0.105 ^de^	15.3
Pusa 3003-15-121-9-5-3	87.1 ^a^	264.2 ^e^	1.13 ^a^	0.12 ^e^	168.3 ^fg^	0.56 ^h^	0.13 ^e^	18.3
Pusa 3003-15-121-9-5-5	84.8 ^ab^	271.0 ^de^	1.10 ^ab^	0.13 ^e^	183.5 ^fg^	0.61 ^gh^	0.15 ^de^	16.3
Pusa 3003-15-121-9-6-3	75.2 ^abc^	418.3 ^ab^	0.97 ^abc^	0.31 ^abc^	333.0 ^bc^	1.21 ^cd^	0.25 ^cde^	5.4
Pusa 3003-15-121-9-7-2	77.0 ^abc^	371.6 ^bcd^	0.99 ^abc^	0.25 ^b–e^	289.7 ^cd^	1.04 ^de^	0.23 ^cde^	7.7
Pusa 3003-15-121-30-23	69.4 ^bc^	426.1 ^ab^	0.90 ^bc^	0.32 ^ab^	360.7 ^abc^	1.38 ^bc^	0.31 ^cd^	4.3
Pusa 3003-15-121-31-1	50.1 ^d^	407.4 ^ab^	0.65 ^d^	0.30 ^a–d^	379.5 ^ab^	1.66 ^a^	0.50 ^b^	3.1
Pusa 3003-15-121-9-27	62.6 ^cd^	436.8 ^ab^	0.81 ^cd^	0.34 ^ab^	388.9 ^ab^	1.56 ^ab^	0.37 ^bc^	2.7
Pusa 44	87.5 ^a^	263.2 ^e^	1.13 ^a^	0.12 ^e^	165.7 ^g^	0.54 ^h^	0.12 ^e^	19.1
IR90019-22-28-2-B	33.6 ^e^	356.4 ^b–e^	0.43 ^e^	0.23 ^b–e^	349.1 ^bc^	1.70 ^a^	0.66 ^a^	4.0
CV%	5.1	7.2	5.2	16.7	6.7	6.5	16.1	-
CD (5%)	15.8	101.8	0.2	0.15	73.3	0.26	0.16	-

RTOL, relative stress tolerance; GMP, geometric mean productivity; SSI, stress susceptibility index; STI, stress tolerance index; HAM, harmonic mean; YI, yield index; YSI, yield susceptibility index. Rank sum is the sum of individual ranks for different tolerance index for each line. Average rank is the mean of ranks of all the stress response parameters. Correlations between average rank and grain yield under stressed and unstressed conditions were −0.958 and 0.262, respectively. Means followed by similar letters are not statistically significant at 5% level, based on Tukey’s honestly significant test.

**Table 7:** Grain quality data for Pusa 44 *qDTY12.1* NILs under stressed and unstressed treatments.

Pedigree	KLBC		KBBC		LBR		KLAC		ER	
	S	US	S	US	S	US	S	US	S	US
Pusa 3003-15-121-17-47-2	5.95 ^b^	6.88 ^a–e^	2.09 ^ab^	2.05	2.85 ^ab^	3.36	10.3 ^b^	10.5 ^c–g^	1.73 ^abc^	1.52 ^c^
Pusa 3003-15-121-17-47-5	5.59 ^cde^	7.15 ^abc^	1.96 ^b^	1.93	2.84 ^ab^	3.70	9.4 ^def^	11.3 ^a–e^	1.68 ^bc^	1.58 ^abc^
Pusa 3003-15-121-29-2-1	5.23 ^fg^	6.56 ^de^	2.01 ^ab^	2.06	2.59 ^b–e^	3.21	9.3 ^ef^	10.4 ^d–g^	1.75 ^abc^	1.58 ^abc^
Pusa 3003-15-121-29-21-4	5.05 ^g^	6.62 ^cde^	2.01 ^ab^	2.12	2.52 ^de^	3.14	9.1 ^ef^	10.5 ^c–g^	1.81 ^ab^	1.59 ^abc^
Pusa 3003-15-121-29-23-2	5.41 ^def^	6.64 ^b–e^	2.04 ^ab^	2.18	2.64 ^b–e^	3.06	9.3 ^def^	10.3 ^efg^	1.72 ^abc^	1.55 ^bc^
Pusa 3003-15-121-29-25-2	5.30 ^efg^	6.67 ^b–e^	1.98 ^b^	2.02	2.67 ^b–e^	3.31	8.9 ^f^	10.8 ^b–g^	1.67 ^bc^	1.62 ^abc^
Pusa 3003-15-121-29-3-1	5.12 ^fg^	6.97 ^a–e^	1.97 ^b^	2.06	2.60 ^b–e^	3.39	8.9 ^f^	11.5 ^abc^	1.73 ^abc^	1.64 ^abc^
Pusa 3003-15-121-29-32-3	5.16 ^fg^	7.26 ^a^	2.04 ^ab^	2.06	2.52 ^de^	3.51	9.2 ^def^	11.7 ^ab^	1.79 ^abc^	1.61 ^abc^
Pusa 3003-15-121-31-32	5.22 ^fg^	6.91 ^a–e^	2.04 ^ab^	2.10	2.56 ^cde^	3.29	9.5 ^cde^	12.1 ^a^	1.83 ^a^	1.75 ^a^
Pusa 3003-15-121-9-37-2	5.28 ^efg^	6.75 ^a–e^	2.01 ^ab^	2.02	2.62 ^b–e^	3.36	9.4 ^def^	11.0 ^b–g^	1.77 ^abc^	1.62 ^abc^
Pusa 3003-15-121-9-43-2	5.34 ^d–g^	6.67 ^b–e^	1.96 ^b^	2.01	2.72 ^b–e^	3.32	9.5 ^cde^	10.2 ^fg^	1.77 ^abc^	1.53 ^c^
Pusa 3003-15-121-9-5-3	5.42 ^def^	7.06 ^a–d^	2.01 ^ab^	2.04	2.70 ^b–e^	3.48	9.5 ^cde^	12.3 ^a^	1.75 ^abc^	1.74 ^ab^
Pusa 3003-15-121-9-5-5	5.43 ^def^	6.48 ^e^	1.98 ^b^	1.92	2.73 ^b–e^	3.38	9.7 ^cd^	10.1 ^g^	1.79 ^ab^	1.56 ^abc^
Pusa 3003-15-121-9-6-3	5.42 ^def^	6.81 ^a–e^	2.00 ^ab^	2.04	2.71 ^b–e^	3.35	9.8 ^bcd^	10.8 ^b–g^	1.81 ^ab^	1.58 ^abc^
Pusa 3003-15-121-9-7-2	5.44 ^def^	7.17 ^ab^	2.00 ^ab^	2.10	2.71 ^b–e^	3.42	9.5 ^cde^	11.7 ^ab^	1.75 ^abc^	1.64 ^abc^
Pusa 3003-15-121-30-23	5.65 ^bcd^	6.89 ^a–e^	2.04 ^ab^	2.05	2.76 ^bcd^	3.37	10.0 ^bc^	11.4 ^a–d^	1.77 ^abc^	1.66 ^abc^
Pusa 3003-15-121-31-1	5.59 ^cde^	6.83 ^a–e^	2.12 ^ab^	2.13	2.64 ^b–e^	3.21	9.6 ^cde^	11.3 ^a–f^	1.71 ^abc^	1.65 ^abc^
Pusa 3003-15-121-9-27	5.81 ^bc^	6.67 ^b–e^	2.08 ^ab^	1.93	2.79 ^bc^	3.47	9.6 ^cde^	10.9 ^b–g^	1.65 ^c^	1.63 ^abc^
Pusa 44	5.06 ^g^	6.83 ^a–e^	2.04 ^ab^	2.03	2.49 ^e^	3.37	9.2 ^def^	10.8 ^b–g^	1.83 ^a^	1.58 ^abc^
IR90019-22-28-2B	4.93 ^h^	5.44 ^f^	1.62 ^c^	1.91	3.04 ^a^	3.26	11.1 ^a^	10.1 ^g^	1.70 ^abc^	1.54 ^bc^
CV%	1.57	1.98	1.89	4.04	2.36	4.19	1.44	2.37	1.91	3.04
CD (5%)	0.34	0.54	0.15	ns	0.25	ns	0.55	1.05	0.13	0.19

KLBC, kernel length before cooking, in mm; KBBC, kernel breadth before cooking, in mm; L/B, length/breadth ratio; KLAC, kernel length after cooking, in mm; KBAC, kernel breadth after cooking, in mm; ER, elongation ratio; HULL, hulling recovery percent; MILL, milling recovery percent; ns, non-significant; CV%, coefficient of variation, CD, critical difference; S, stressed; US, unstressed. Means followed by similar letters are not statistically significant at 5% level, based on Tukey’s honestly significant test.

## Data Availability

Data used in this study are presented in the supplementary information.

## Supplementary Materials

The following are available online at https://www.mdpi.com/article/10.3390/genes12070967/s1, Table S1: Agronomic characteristics of Pusa 44 *qDTY12.1* near isogenic lines during *Rabi* 2018–2019 at Aduthurai.

## Abbreviations

ANOVA	Analysis of variance
BC	Backcross
bp	Base pairs
BS	Background selection
CD	Critical difference
CmN	Number of tillers (culms)
CV	Coefficient of variation
DFF	Days to 50% flowering
ER	Elongation ratio
FS	Foreground selection
g.sqm^−1^	Grams per square meter
GMP	Geometric mean productivity
GQ	Selection for grain and cooking qualities
GY	Grain yield
HAM	Harmonic mean
Ht	Plant height
HULL	Hulling recovery
IARI	Indian Agricultural Research Institute
ICAR	Indian Council of Agricultural Research
KER	Kernel elongation ratio
KLAC	Kernel length after cooking
KLBC	Kernel length before cooking
kPa	Kilo Pascal
LBR	Length by breadth ratio
MABB	Marker assisted backcross breeding
MILL	Milling recovery
NIL	Near isogenic line
PnL	Panicle length
PS	Phenotypic selection
qDTY	QTL for yield under drought
QTL	Quantitative trait loci
RBGRC	Rice breeding and genetics research centre
RM	Rice microsatellite
RP	Recurrent parent
RPG	Recurrent parent genome
RPP	Recurrent parent phenome
RPGR	Recurrent parent genome recovery
RSDS	Reproductive stage drought stress
RTOL	Relative stress tolerance
S	Stressed
SF	Spikelet fertility
SSI	Stress susceptibility index
SSR	Simple sequence repeats
STI	Stress tolerance index
t.ha^−1^	tonnes per hectare
US	Unstressed
YI	Yield index
Yld	Grain yield per plot
YSI	Yield stability index
