# Supplementary material for: Molecular Breeding for Improving Productivity of Oryza sativa L. cv. Pusa 44 under Reproductive Stage Drought Stress through Introgression of a Major QTL, qDTY12.1
Source: Genes (Basel). 2021 Jun 24;12(7):967. doi: 10.3390/genes12070967 (PMC8303740; doi:10.3390/genes12070967)
Supplement: Supplementary file 1 [file genes-12-00967-s001.zip › genes-1126975-supplementary.pdf]

Supplementary Table S1. Agronomic characters of Pusa 44 *qDTY12.1* near isogenic lines during 2019 dry season in Aduthurai.

No.	Pedigree	DFF (days)	PH (cm)	TLN	PL (cm)	FGNP	TGP	SFP (%)	1000GW (g)	PLY (g.sqm^{-1})
1	Pusa 3003-15-121-17-47-2	108.50	90.50^{ab}	9.9	23.05^{b}	128.10^{ab}	167.00^{ab}	76.64^{a}	20.52^{ab}	400.50^{ab}
2	Pusa 3003-15-121-17-47-5	114.50	91.60^{ab}	13.6	23.50^{ab}	124.10^{ab}	180.40^{ab}	69.64^{a}	20.39^{abc}	412.84^{ab}
3	Pusa 3003-15-121-29-2-1	108.50	80.20^{cd}	15.3	24.92^{ab}	94.30^{b}	164.20^{b}	57.42^{a}	19.31^{b-e}	294.33^{b}
4	Pusa 3003-15-121-29-21-4	110.00	87.20^{a-d}	11.8	25.23^{ab}	123.10^{ab}	203.70^{ab}	61.30^{a}	18.80^{de}	473.34^{ab}
5	Pusa 3003-15-121-29-23-2	109.50	88.00^{a-d}	12.0	24.87^{ab}	127.20^{ab}	187.80^{ab}	67.47^{a}	18.76^{de}	392.16^{ab}
6	Pusa 3003-15-121-29-25-2	109.50	88.00^{a-d}	10.5	24.98^{ab}	133.80^{ab}	197.90^{ab}	67.64^{a}	20.23^{abc}	392.16^{ab}
7	Pusa 3003-15-121-29-3-1	108.00	90.90^{ab}	16.9	25.43^{ab}	144.60^{ab}	195.40^{ab}	72.80^{a}	19.24^{b-e}	411.66^{ab}
8	Pusa 3003-15-121-29-32-3	107.00	92.90^{a}	11.3	24.30^{ab}	133.90^{ab}	188.10^{ab}	71.05^{a}	20.25^{abc}	369.34^{ab}
9	Pusa 3003-15-121-31-32	108.00	87.50^{a-d}	11.3	24.04^{ab}	131.30^{ab}	173.70^{ab}	75.38^{a}	21.09^{a}	377.66^{ab}
10	Pusa 3003-15-121-9-37-2	109.00	93.80^{a}	13.8	24.32^{ab}	129.10^{ab}	201.20^{ab}	65.84^{a}	19.44^{b-e}	352.66^{ab}
11	Pusa 3003-15-121-9-43-2	108.50	90.90^{ab}	12.9	24.53^{ab}	136.50^{ab}	223.80^{a}	60.49^{a}	19.24^{b-e}	361.00^{ab}
12	Pusa 3003-15-121-9-5-3	107.00	88.50^{abc}	11.4	24.20^{ab}	137.30^{ab}	204.20^{ab}	67.50^{a}	19.16^{b-e}	327.52^{b}
13	Pusa 3003-15-121-9-5-5	108.00	89.00^{abc}	16.7	25.82^{a}	163.50^{a}	225.20^{a}	72.61^{a}	18.71^{de}	279.50^{b}
14	Pusa 3003-15-121-9-6-3	107.00	91.00^{ab}	10.1	25.56^{a}	156.30^{ab}	211.50^{ab}	74.47^{a}	19.73^{a-e}	476.72^{ab}
15	Pusa 3003-15-121-9-7-2	108.00	86.40^{a-d}	13.0	24.69^{ab}	96.30^{b}	178.40^{ab}	54.05^{a}	19.62^{b-e}	327.67^{b}
16	Pusa 3003-15-121-30-23	108.00	91.20^{ab}	12.3	24.69^{ab}	125.40^{ab}	181.80^{ab}	69.28^{a}	19.80^{a-d}	540.00^{a}
17	Pusa 3003-15-121-31-1	108.00	78.40^{d}	19.4	24.51^{ab}	112.20^{ab}	161.70^{b}	70.48^{a}	19.02^{cde}	322.67^{b}
18	Pusa 3003-15-121-9-27	108.00	83.90^{a-d}	13.1	24.94^{ab}	135.90^{ab}	195.70^{ab}	70.62^{a}	19.24^{b-e}	400.50^{ab}
19	Pusa 44	108.00	82.50^{bcd}	9.2	25.20^{ab}	148.00^{ab}	192.20^{ab}	76.26^{a}	18.34^{e}	402.67^{ab}
	CV%	1.67	2.78	20.87	2.45	12.16	7.54	8.43	1.79	13.54
	CD	ns	9.92	ns	2.45	64.45	58.55	23.44	1.42	211.66

DFF, Days to 50% flowering; PH, Plant height; TLN, tillers number per hill; PL, panicle length; FGNP, filled grains number per panicle; TGP, number of total grains per panicle; SFP, spikelet fertility per cent; 1000GW, 1000 grain weight; PLY, plot yield, ns, non-significant; CV%, coefficient of variation; CD, critical differences,
